# A neo-institutional analysis of the hidden interaction between the Israeli Supreme Court and the Ministry of Finance: the right to healthcare services

**DOI:** 10.1186/s13584-018-0261-9

**Published:** 2018-11-27

**Authors:** Daniel Sperling, Nissim Cohen

**Affiliations:** 1 Department of Master Degree Studies in Health Systems Management, Yezreel Valley Academic College and Faculty of Law, University of Haifa, Haifa, Israel; 2 Department of Public Administration & Policy, University of Haifa, Haifa, Israel; 3 Department of Health Systems Management, Yezreel Valley Academic College, 19300 Yezrael Valley, Israel

**Keywords:** Health law, Economic arrangements law, Right to healthcare services, Judicial review, Supreme court, Neo-liberalism, Institutional analysis

## Abstract

**Background:**

Under structural conditions of non-governability, most players in the policy arena in Israel turn to two main channels that have proven effective in promoting the policies they seek: the submission of petitions to the High Court of Justice and making legislative amendments through the Economic Arrangements Law initiated by the Ministry of Finance. Nevertheless, an analysis of the principal trends emerging from the High Court of Justice rulings and legislative amendments through the Economic Arrangements Law indicates that these channels are open to influence, primarily by forces that are essentially neo-liberal. Little is known about the effects of these trends on the right to healthcare services, which in Israel has not been legislated as an independent constitutional law in Basic Laws.

**Methods:**

We use four major legal cases decided by the Supreme Court of Israel in the past 10 years where the Court reviewed new legislative initiatives proposed by the Economic Arrangements Law in the area of healthcare. We utilize an institutional approach in our analysis.

**Results:**

A neo-institutional analysis of the legal cases demonstrates that petitions against the Economic Arrangements Law in the area of healthcare services have been denied, even though the Court uses strong rhetoric against that law and the government more generally in addressing issues that concern access to healthcare services and reforms in the healthcare system. This move strengthens the trend toward a neo-liberal public policy and significantly weakens the legal protection of the right to healthcare services.

**Conclusion:**

In deciding petitions against the Economic Arrangements Law in the area of healthcare, the Supreme Court allows the Ministry of Finance to be a dominant player in the formation of public policy. In doing so, it may be promoting a goal of strengthening its position as a political institution that aspires to increase the public’s trust in the judiciary and especially in the Supreme Court itself, in addition to exercising judicial restraint and allowing more leeway to the executive and legislative branches more generally.

**Electronic supplementary material:**

The online version of this article (10.1186/s13584-018-0261-9) contains supplementary material, which is available to authorized users.

## Introduction

For three decades now, Israeli society has faced a variety of institutional changes that have significantly altered the nature of the Israeli welfare state and its healthcare system. The difficult problems of non-governability, namely, the inability of society’s decision makers, politicians, and bureaucrats to shape public policy and implement it on the ground effectively, facing Israeli society [1–4] help explain these changes and how they take place. While these problems started in the 1970s with a growing tension between the government and society, they reached their peak at the beginning of the twenty-first century with increasing social and economic gaps among constituent groups in society. As a result, there has been a decline in the politicians’ power and their ability to overcome the various demands of self-serving interest groups [5].

The literature has recently acknowledged that when it comes to providing services, the Israeli government does not provide enough support in terms of either quantity or quality. Numerous structural conditions and social processes have led large swaths of Israeli society to make it a rule of thumb to “create facts on the ground.” This activity, which has been termed “alternative politics,” is evident in the literature on Israel in general [6] as well as specifically in the Israeli healthcare policy arena [7, 8]. The term includes increased appeals to alternative channels, many times in the private sector, in order to provide services more rapidly than the government can manage.

Under the structural conditions of ongoing non-governability [2], most players in the policy arena turn to two main channels that have been proven effective: the submission of petitions to the High Court of Justice and making legislative amendments through the Economic Arrangements Law (hereinafter: “the Law”). These two channels have received a great deal of criticism in the literature and public discourse. Analysis of the principal trends emerging from High Court of Justice rulings and legislative amendments through the Economic Arrangements Law indicates that these channels are open to influence, primarily by forces that are essentially neo-liberal.

As in other societies around the world [9, 10], scholars in Israel point to a radical ideological shift in Israel’s social policy. Hence, Bareli et al. [11] argue that a significant ideological shift among decision-makers, especially politicians, is the primary cause of the decline of the welfare state. Since the late 1990s, Israeli governments have adopted socio-economic policies that exhibit clear neo-liberal characteristics. Indeed, along with local and global economic factors and Israel’s national security problems, the politicians *are* part of this reality.

However, this article highlights another significant reason for the current situation. Rather than the ideological gap between Israeli decision makers and the public leading to the current policy [12], we maintain that over time, institutional changes have created a reality in which the nature of the interaction between Ministry of Finance and the Israeli Supreme Court has changed. Specifically, we argue that the nature of the relations between the Ministry of Finance bureaucrats, the key players in legislation through the Law of Economic Arrangements, and the Supreme Court, where petitions challenging this legislation are heard, is such that the Supreme Court strengthens the position of the Ministry of Finance as a dominant player in the formation of public policy. This outcome has occurred as the result of two strategies. Using the first strategy, the court seeks to increase public trust in the judiciary and improve its standing with a wide variety of sectors within the population (including those who support the welfare state perspective) at the cost of political friction and active intervention in the activities of other authorities. Such a strategy usually takes part outside the health or social rights contexts, most notably with regard to cases that involve the constitutional rights to liberty and property. Examples for this strategy can be seen in Ruling 10042, 10046, 10054/16 and 76, 802/17 [13] where the Court invalidated part of the Economic Arrangements Law that levied special tax on owners of more than two apartments finding that Knesset members have not fully participated in the parliamentary process and in Ruling 8260/16 [14] where the Court ruled that the Knesset is barred from enacting for the sixth time a temporary law on biennial budget that would have violated Basic Law: The State Economy (1975).

Using the second strategy, the Court still acts to increase public trust in the Supreme Court but compromises the level of trust in a way that does not have a negative impact on its relations with other political institutions, specifically its relationship with the Finance Ministry. The result of this move is the strengthening of the position of the Court as a political institution that aspires to increase the public’s trust in the judiciary, especially in the Supreme Court itself. We therefore focus our study on the role of the Supreme Court in this interaction.

Moreover, given that the public sees only case decisions, the interaction between these two institutions seems to be hidden from the public eye, and in some cases is masked as an “ordinary” political interaction between the Supreme Court and the Knesset (the Israeli parliament). Thus, social-democratic forces are relegated to working through “regular” legislative means, which have been proven to be less effective in light of existing structural conditions. The results of these institutional developments include the strengthening of the trend toward a neo-liberal public policy, the decline of the welfare state, the privatization of the healthcare system in Israel, and a significant weakening of the legal protection of the right to healthcare services.

As the article will show, the Israeli case helps explain how, through the function of judicial review, the Judiciary seeks to increase its institutional power relative to the Government. It also reveals the effects that an enabling strategy allowing the Ministry of Finance bureaucrats to be the key player in the formation of policy have on the legal protection of the right to healthcare services. Moreover, as we will describe, changes in Israeli attitudes towards the welfare state in recent decades parallel developments in the US since the 1970s. The “hostility” that became the predominant ideology in Israel mirrored the acceleration of the conservative ideology about government and social welfare in the US in the 1980s. Furthermore, the Israeli problem of non-governability that created the public perception that the government could no longer “deliver the goods” is a problem not completely unfamiliar to the US and other Western democracies. This issue is also echoed in the description of the gridlocked Congress or governments with divided party control and social polarization [15, 16]. A recent example is the ongoing threat of repealing President Obama’s national healthcare reform in the US. Such a threat has a negative impact on the implementation of this new policy by causing a “wait and see” attitude among US healthcare leaders, characterizing systems with gridlock. Thus, while our analysis focuses on the Israeli case, the Israeli experience has several implications for other democratic countries – especially those characterized by a significant gap between their social policy in practice and public satisfaction with it [12].

Furthermore, our analysis can also be generalized to other political systems where the courts (especially the Supreme Court) seek to increase their institutional power and legitimacy. While the literature regards judicial review as a legitimate forum for resolving the competing interests of the Court and the Parliament [5, 17] with a negligible influence of the Court on politics and policy [18], aside from more general explanations concerning judicial restraint the analysis we provide reveals how the Supreme Court seeks to increase its institutional power at the expense of the Government in an era of non-governability. The result of these efforts may be the abdication of the Court’s responsibility to protect the right to healthcare services to the Ministry of Finance (rather than the Knesset). As our article will show, the various outcomes that support this conclusion reflect a real departure from what is currently discussed within Israeli politics.

### Social institutions and the new institutional approach

The institutional literature has had a strong influence on the analysis of public policy and the welfare state [9, 10, 19, 20]. Social institutions are designed to help people cope with the daily problems of life within society. The broadest and most agreed-upon definition of the term “social institution” relates to constraints or the rules of game leading to stability in the relations between human beings. Thus, institutions include conventions as well as official and unofficial norms [21]. North [22] defines an institution as “the rules of the game” (official or unofficial). Institutional arrangements are the result of political struggles and power relations among organized groups during a window of opportunity for action [23]. Such institutions can ensure consistency with respect to individuals’ expectations, which is a precondition for institutional balance or stability. As we shall see below, the authority of the High Court of Justice and the legal status of the Law of Economic Arrangements indicate that both structures are formal social institutions.

New institutionalism suggests two main approaches for analyzing institutional change: radical and incremental transformative change. Incremental and gradual changes may be minor ones that adapt or re-shape the existing institution or major ones that completely revamp the institution and therefore are transformative in their nature [9]. The second approach relies on a strong punctuated equilibrium model where long periods of institutional stability are interrupted by some sort of exogenous shock or crisis leading to a more or less radical reorganization followed by institutional stability ([24]; Katzenelson and Weingast, 2005; [25]).

In this article we will adopt an institutional approach through which we will show that, when reviewing the Law of Economic Arrangements, the Israeli Supreme Court may be actually formulating policy changes that fuel and reinforce its status as a political institution. The policy change on which we focus is related to rulings affecting the formulation of the legal right to healthcare services and the Court’s willingness to interpret it in a way that would have regarded it as linked to the explicit constitutional rights acknowledged in the Basic Law: Human Dignity and Liberty. Defining privatization in its broad sense, as the act of reducing the role of government or increasing the role of private institutions in satisfying people’s needs [26], we claim that such rulings may increase privatization trends in the healthcare system, and, more broadly, contribute to the decline of the welfare state.

### The economic arrangements law in Israel as a social institution

Many of the legislative changes in the National Health Insurance Law were made by a series of Economic Arrangements Laws [21]. The first Economic Arrangements Law was passed in 1985, as part of a plan to stabilize the economy. Because of the extremely difficult situation in the Israeli economy (especially high inflation rates, a deepening budgetary deficit and diminishing monetary reserves alongside a crisis in the financial and banking system following the stock market crisis of 1983) ([27]: 9; [28]), an emergency plan, which in part clashed with various agreements and even laws on a variety of policy issues, was adopted. The law is unique in that politicians are required to vote on each of its provisions. However, it must pass as a single piece of legislation, even though it covers various policy domains and issues (e.g., health, education, transportation) [5]. Since its passage as emergency legislation, the Law of Economic Arrangements has become an accepted practice, brought before the Knesset for approval alongside the debate over the Budget Law (at least 60 days before the end of the fiscal year) or as part of the government’s economic plan.^1^

The legislation of the Law of Economic Arrangements has unique characteristics [29]. The law comprises a variety of issues, which serve as the means of policy implementation or transformation, including adjusting, suspending or eliminating existing legislation.^2^ The legislative initiative comes from the Ministry of Finance bureaucrats (in contrast to governmental legislative bills initiated by the responsible ministry or by the Ministry of Justice). The debate over the range of issues is usually conducted en masse and in an expedited process. The legislative bill usually includes many paragraphs and pages (over 100 pages, for example, in the 2004 bill). Most of its important issues are turned over to the Finance Committee for debate (rather than being divided up for debate among the professional and issue-related committees of the Knesset),^3^ where the government usually has an almost automatic majority.^4^ The Law may be described as a significant factor affecting the judgment of decision makers, the political and economic power bases, and the degree of internalization of democratic norms within the political and public system of Israel ([27]: 8).

The rationales for passing this legislation can certainly be used to help us understand the activities of a key player in the arena of healthcare policy – the Ministry of Finance bureaucrats.^5^ This institutional arrangement was originally created because of circumstances of extreme non-governability in the Israeli political system [4] and in the context of a political culture characterized by the circumvention of formal institutional channels ([5]: 303). Focusing on the building of a hospital in Ashdod as a case study, Cohen [30] argues that the Israeli healthcare system demonstrates the non-governability that characterizes most of the policy domains in Israel. The frequent changes in government not only challenge the possibility of a long-term, strategic healthcare policy design in Israel, but also motivate senior bureaucrats to bypass the Knesset and turn to other channels. As a senior bureaucrat in the Ministry of Health explained: “The regular legislation is terribly frustrating, and you say – why should I go for it? If I overcome the obstacle of the Ministry of Finance, the story is over… This way [using the Economic Arrangements Law, *the authors*] it is possible to get things done within 7-8 months. If we used regular legislation, it would take two or three years. Imagine that each time a minister is replaced, a government collapses or a committee chairman is replaced, everything needs to be started again…” (in: [30]: 651).

By means of this law, the government seeks to advance a neo-liberal ideology, aiming to transfer many government functions to the free market and limit the government’s authority to enforce economic rights and basic liberties. Since the passage of the National Health Insurance Law and up to October 2009, it has been subjected to 416 different amendments. Two hundred and eighty of these amendments were legislated through the “Health Chapter” of the Law of Economic Arrangements and in the context of laws aimed at Israel’s economic recovery [31]. Through these legislative amendments, the goal of privatizing the healthcare system has been realized and paradoxically, been identified with the goal of accelerated centralization and regulation of the Israeli healthcare system on the part of the government and Ministry of Finance [31]. Because of the unique mechanism of the Law, the Ministry of Finance was able to bring about fundamental and speedy changes to the principles of the National Health Insurance Law. These changes have undermined its foundational idea expressed in Paragraph 1 of the law, according to which national health insurance would be based on principles of justice, equality, and mutual assistance.

### The judicial critique of the law of economic arrangements

While in principle the Supreme Court has recognized its own authority to intervene in the legislative process [32], it will usually not review the Knesset’s legislative procedures, including that of the Law of Economic Arrangements, where they have not been terminated [33].^6^ This rule derives from the principle of separation of powers and the idea that the Court seeks to respect the legislative body and allow it to exert its sovereign authority as a representative of the public [34]. The Court recognizes its own authority to intervene in the legislative process of the Law of Economic Arrangements in order to hear arguments about its constitutionality, but only after the conclusion of the legislative procedures ([35]: 97).^7^

Indeed, judicial intervention in legislative activity regarding socio-economic policy is not taken lightly. In general, the Court will exercise self-restraint when it comes to intervention in Knesset activity [36]. The rationale for this restraint is that, given the reality of limited resources, a ruling that obligates the state to allocate resources for a certain socio-economic objective will necessarily come at the expense of other objectives, which might also be important.

Some also claim that the parliamentary forum that represents various interest groups is the most appropriate venue, certainly in comparison to the Court, for resolving these questions [37]. According to this perspective, making budgetary decisions about social goods is a political activity and should be made by representatives of the public.^8^

More specifically, in *the matter of the Poultry Breeders Organization* [29], the Supreme Court noted that the Law makes a probing and comprehensive debate very difficult, which, in turn, undermines the ability of decision makers in the government and Knesset to adopt an informed stance on each of the issues covered in the proposed Law. This outcome is contrary to the objectives of the Knesset’s constitution, which states that Knesset members should be allowed to formulate a stance in a calm and collected manner on every legislative issue placed before them ([29]: 33–4). Moreover, such a process does not enable the public monitoring and oversight of legislators, government ministers, or Knesset members (of its various committees), making it possible for the government to avoid effective critique of its initiatives and upsetting the delicate balance between government branches.^9^ Thus, the Court determined that in those extreme and rare cases where the impact of the Law would be so great, and the legislative procedure would be so hasty, the Court would have no alternative but to declare that the Law prevented Knesset members from having a substantial opportunity to make up their mind on the Law. Therefore, a serious and clear violation of the principle of representation had occurred ([29]: 49–50).

Indeed, following Supreme Court judgements showing dissatisfaction from the legislation process of Economic Arrangements Laws, the Knesset, through its Speaker and Legal Advisor, better control the power of the Government to pass these laws. This is done by demonstrating greater involvement in negotiating with the Ministry of Finance and the Government before the first reading of these laws in the Knesset about the issues that would be included in these laws. As a result, some of the issues are totally excluded from such a legislation if, for example they are not related to the budget or are not ripe for legislation. In these negotiations it can also be agreed that some other issues would be included in the legislation after the first reading. Only then would these issues be discussed within the different committees of the Knesset. Among these issues, some may also be routed to the regular process of legislation and not to the special process that is linked to the approval of the state budget.

### The supreme court as a political institution

This section will focus on one explanation for understanding the dynamics between the Court and Ministry of Finance through institutional analysis of judicial review of the Law of Economic Arrangements in the area of healthcare. According to the Theory of Moves [38], which analyzes reciprocal relations among various players, it is reasonable to assume that as a political institution aspiring to increase its power, the Supreme Court has two potential strategies when reviewing the Law initiated by the Finance Ministry. The first is to seek increased public trust in the judiciary and improve its standing with a wide variety of sectors within the population (including those who support the welfare state perspective) at the cost of political friction and active intervention in the activities of other authorities. The second strategy is to still act to increase public trust in the Supreme Court, but to compromise the level of trust in a way that does not have a negative impact on its relations with other political institutions, and specifically for our purposes, its relations with the Finance Ministry. Both of these strategies are driven by the desire of the Supreme Court to increase its power and public support. Moreover, they go hand in hand with and reflect a neo-liberal ideology that, as argued above, supports the protection of the value of liberty by severely criticizing the foundations of equality, thereby strengthening the ties between liberty and private ownership. While the second course of action promotes such a philosophy more directly, under the first strategy the Court seeks to appear more neutral with regard to its ideology. The Court’s assumption here is that occasional political friction with the Ministry of Finance would increase its institutional power and its perception as an objective and apolitical institution.

We contend that, beginning in the mid-1990s, and alongside broader transformations that affected its judicial intervention, the Supreme Court of Israel operated in a way that fueled, supported, and affirmed the Finance Ministry through the mechanism of the Law of Economic Arrangements. This article focuses on one possible motivation for doing, that is to advance its own political and institutional interests, which had been reformulated during this time. In so doing, the Court promoted values that are at the heart of the libertarian neo-liberal ideology, a philosophy that does not accept the substantive changes that took place within liberalism in the twentieth century. This change involved the favoring of the value of equality over the value of liberty and the perspective that liberty derives from private ownership.^10^ Rather, this philosophy seeks to bring back the primacy of the value of liberty by severely criticizing the foundations of equality in the liberal perspective and strengthening the ties between liberty and private ownership. Accordingly, such an approach sanctifies such values as autonomy, free competition, private ownership, and efficiency, and holds that the redistribution of wealth within society violates the proprietary rights of the individual.^11^ An example of this philosophy is the Supreme Court’s resistance to recognizing constitutional standing for social rights (and for our purposes, the right to healthcare services) or to see them as part of “human dignity” as embodied in the Basic Law: Human Dignity and Liberty [39–41].^12^

Nonetheless, in a number of cases submitted to the Supreme Court in recent years that dealt with the appropriate scope of the Law of Economic Arrangements with regard to healthcare, a certain change in the Court’s rhetoric could be discerned. This change reflects the Court’s criticism not only of the hasty manner in which the Law is passed ([29]: 55–7), but also of the appropriateness of the issues addressed in this legislation. This issue is of particular concern given the extensive use that the government seeks to make of this law [42, 43]. This change increases political friction between the Court and the Finance Ministry, which initiates the Law, and between the Court and the Knesset, which approves it. Nevertheless, the Court continues to support and reinforce the position of the Finance Ministry by almost automatically repeating its general rule that it will not intervene in the Knesset’s legislative process before it is completed. For the most part, the Court does not find that the cases heard are “one of those cases where the government grossly exceeded the broad range of reasonability and proportionality granted to it for purposes of legislation that would justify judicial intervention in government discretion before the procedures in question have been completed” [44].

The starting point of the institutional analysis above is that the Supreme Court is an active player in the determination of public policy and one of the strongest institutions and political players. Although it is limited to the legal and institutional framework that determines its relationship with other institutionally powerful players, the Court does seek to promote its own interests. However, like any other policy actor, the judges are also limited by public opinion, and tend to rule in line with public sentiment reflected in social struggles and political pressure, when the law is broad enough or flexible enough to allow it ([45], p. 72). Indeed, in recent years the Court has at times ruled in favor of vulnerable populations such as prisoners who petitioned against their spacious conditions in jail [46], asylum seekers from Sudan and Eritrea who were subject to detention [47] and parents to Palestinian students in Eastern Jerusalem having to pay out-of-pocket expenses for the education of their children due to shortage in classes in public schools in Eastern Jerusalem [48]. Regardless of these relatively small number of cases, some sociolegal theories of law hold that judges in general, and the Supreme Court in particular seek to be accepted by the public, especially those elements that empower them as political bodies, namely, the middle and upper classes, and the relevant legal community associated with them ([45], p. 72; [49], p.26). In a pioneering study, Mizrahi and Meydani [Editor’s note: The date is missing] demonstrated that, since the mid-1980s, there has been a visible increase in the standing of the Court. One of the explanations for this increase is the practice of politicians and legislators who were unable to govern effectively to turn to the Supreme Court and request its intervention in the activities of these authorities. The Court’s increased power is expressed in its repeal of Knesset legislation, the expanded range of issues that it reviews, and the broadening of the right of standing of petitioners. It also seeks to strengthen public trust in the judicial authority ([50–53]: 53) and reinforce its judicial legitimacy through public support [54], which, paradoxically, has declined over the years [55, 56].

Indeed, the Court’s desire to increase the public’s trust in it is just one of many factors that may explain its limited intervention in issues pertaining to the Law of Economic Arrangements dealing with social questions in general and healthcare matters in particular. These factors may be associated with the weak constitutional status of social rights [37, 57], the relative inferiority of social rights to civil and political rights) [58, 59]), the difficulty and reluctance of the courts to review socio-economic and policy issues that have significant budgetary implications [57], the personal and social backgrounds of the judges [60], who do not necessarily show understanding of, solidarity with or a special empathy for the disadvantaged segments of society [61, 62], and the court’s limited independence, ability or desire to lead fundamental social changes [63].

However, since this article takes a neo-institutional approach to analyzing the hidden interaction between the Supreme Court and the bureaucrats of the Ministry of Finance in reviewing petitions against the enactment of the Law of Arrangements, it will focus on the concept of public trust, which best describes the institutional interests of both players. While other explanations for the Court’s unwillingness to interfere with the Law of Arrangements may refer to more general reasons for the Court’s self-restraint, using the concept of public trust helps focus on the institutional analysis of the Law of Arrangements as a unique model of policy-making rooted in the problem of non-governability characterizing Israeli politics. This approach also illustrates how, despite its neo-liberal ideology, the Court may wish to consider a course of action based on its institutional self-interest in being regarded as an apolitical and objective institution. Unlike other analyses of the welfare state in Western society [9, 10], we maintain that the Supreme Court’s efforts to increase public confidence and strengthen its institutional status in the short term is the most convincing explanation of its interaction with the Israeli Parliament when validating the enactment of the Law of Arrangements.

There are various explanations for the Court’s desire to increase its power as a political institution. From a sociological standpoint, the Court may try to preserve the political and public legitimacy upon which its hegemony rests by increasing the fragmentation and polarization of the legislature and the executive branch [64], and by promoting a gradual shift in liberal values among certain groups of Israeli society, especially the media, and the business and academic communities [65–67]. This position has much in common with the critical legal studies approach, according to which the Supreme Court is regarded as an institution that is part of - and therefore strengthens and legitimizes - the social elite. According to this view, the Court has an interest in preserving the status quo of inequality in society. Doing so allies it with the centers of power that allow it to use various legal strategies such as interpretation and vague concepts to maintain the status quo [68].

According to the institutional perspective that we use, the Supreme Court encourages and provides incentives for various social players such as NGOs, Knesset members and politicians to submit petitions for judicial relief, thereby increasing its institutional interests (along with promoting the social and professional interests of these players). The Court has also developed a quasi-exit mechanism, allowing the public who is not satisfied with and/or does not believe in a specific public policy or cannot act in accordance with it, a policy alternative [69, 70]. In this way, the Supreme Court, together with other social actors, is a catalyst for making policy changes and institutional reforms, driven by clear agendas and ideologies. According to public choice theory, which complements our analysis, the judges have independent interests and goals separate from those of the elite, which stem from their bureaucratic position from which they benefit. The composition of the court, the way judges are appointed (an issue in the US), their worldviews as well as other social and political processes that prompt the Court to interpret the law and approve or reject various policy reforms make the Court a major political institution endowed with a remarkable ability to strengthen its institutional interests [70, 71]. Finally, the media play a role in the increase in the institutional power of the Supreme Court, especially the establishment of its position among the Israeli elite. On one hand, the media have strengthened their image as the democratic watchdog. On the hand, they have also burnished the public image of the Supreme Court as an apolitical and independent institution that has few interests of its own [72].

## Method

We test our contention using four major legal cases decided by the Supreme Court of Israel between 2005 and 2018 years where the Court reviewed new legislative initiatives proposed by the Economic Arrangements Law in the area of healthcare. To prove this contention, we will discuss the very few substantial decisions rulings of the Supreme Court regarding the Law of Economic Arrangements in the healthcare context in the order in which they were made between 2007 and 2016. We utilize an institutional approach in our analysis.

In the matter of *The Society for Patients’ Rights in Israel* et al. [42], the Court heard a petition challenging the Finance Ministry’s proposal under the Law not to allow the inclusion of “life-saving” or “life-extending” medication in the supplementary health services plan and to include the choice of a surgeon without the patient’s participation in that selection. The petition argued that providing life-saving or life-extending medication only to those who chose a supplementary health insurance plan distorts the principles of equality and solidarity at the heart of the National Health Insurance Law. In the petition, the Supreme Court was asked to instruct the Knesset not to hold a second or third reading on the Ministry’s initiative, and alternatively, to instruct the Finance Ministry to remove these initiatives from the legislation.

During the debate over the petition, the Knesset contended that there was no connection between denying the option of including life-saving and life-extending medication in supplemental health services, on the one hand, and the state budget, on the other. Therefore, there was no justification for legislation on this issue in the Law of Economic Arrangements. Nonetheless, it was suggested that the Finance Committee be permitted to debate the proposal as planned, including the question of whether it was appropriate to remove these sections from the Law of Economic Arrangements’ legislation and submit them to regular legislation.

The Court rejected the petition and found that, in accordance with accepted custom,^13^ it cannot intervene in the legislative process while it is ongoing. In doing so, the Court referred to the general ruling regarding judicial intervention in legislative processes. The Court also refrained from commenting on the substantive disconnection between the legislative initiative and the legislation of this Law. However, at the same time, it left open the possible consideration of the matter after the completion of the legislative process. The Court’s decision was short and succinct. It did not address the substantive questions raised in the petition, although it recognized the Court’s authority to reconsider the issue, if requested, after the completion of the legislative process. Another reading of this case suggests that the Court was impressed by the Ministry of Finance’s arguments concerning the violation of the principles of equality and solidarity of having life-saving treatments in the supplemental health insurance policy but not in the basic one.

Another case where the relationship between the Court and Finance Ministry in the healthcare context is evident is the *Israel Medical Association* vs. *the Attorney General of Israel* et al. [43]. This case dealt with a petition to void sections 15 and 16 of the Law of Economic Arrangements of 2006 after its approval in the second and third readings in the Knesset. These sections authorized pharmacists to issue prescription medication without a physician’s prescription. In its petition, the Israel Medical Association argued that the substantive matter of this legislation, namely, the authorization to issue medication, is not among the budgetary issues that the Law is usually meant to address. Furthermore, it contended that this was hasty legislation that did not include a discussion of its far-reaching implications. Finally, the medical association maintained that it was incorporated into the reservations to the Law. This step contradicted the position of the Knesset Committee on Labor, Welfare, and Health, which held that this issue was not to be included in the Law of Economic Arrangements.

The Court (presided over by President Beinisch) recognized that the budgetary element of the legislative amendments was not substantive and that the division of authority between pharmacists and physicians was not among the issues that the Law of Economic Arrangements normally addresses. The Court also noted that the Law had far-reaching implications for the healthcare system and for patients, and that it should have been submitted to the regular legislative channels, which would have included a proper discussion. Thus, the Court did not avoid friction with the Ministry of Finance and the Knesset, and regarded it as proper that it should comment on the appropriateness of this type of legislation. Nevertheless, the Court rejected the petition and, by its ruling, strengthened the Finance Ministry’s position. It noted, per general custom, that, “only flaws that strike at the root of the legislative process and undercut the basic values of our constitutional regime will lead to judicial intervention in the legislative process” ([43]: 4764). Furthermore, the Court found that despite the improper legislative procedures in this case, the resulting flaws were not of the sort that justified its intervention as previously decided in the “Poultry Breeders” case.^14^

Justice Elyakim Rubinstein, who as the Attorney General of Israel instructed the authorities to refrain from the extensive and improper use of the Law ([73], Appendix 6), joined President Beinisch in criticizing the way in which the Law was passed as a matter of principle. In the abovementioned case, Justice Rubinstein addressed the concept of public trust– a concept that may be ascribed to the Court’s demand for institutional political power but which he ascribed to the Knesset. In his words:

Common sense tells us that public trust in the legislative process depends on the seriousness of the process. When the process is practically automatic and when it is hasty or random, the legislator himself develops doubts about the legislation he produces, and considering the price of ongoing loss of public trust, it is doubtful whether the product is worth the damage, that is, whether the achievement of fast and “efficient” legislation of the Law of Economic Arrangements is worth the contempt for the process and accompanying loss of trust… ([43]: 4766).

Justice Rubinstein noted further that this case was very much a borderline case in terms of judicial intervention. He expressed great doubt regarding the question of whether Knesset members who were not part of the Labor, Welfare, and Health Committee had a realistic opportunity to participate in the legislative process to the extent that one could say that the principle of participation as per the “Poultry Breeders” ruling had been upheld. Those doubts held even though the reservation to the Law had formally been made available to all Knesset members and they could have examined it if they wanted to do so. At the conclusion of his decision, Justice Rubinstein added the following words, which are indicative of the Court’s willingness to change its orientation:

This Court is of the view that it is obligated to exercise restraint regarding intervention in legislation and accompanying processes for the good reason of respect among authorities and a variety of other reasons described by my colleague in the Poultry Breeders case (see pp. 53–55). Yet, I foresee a difficult future because as long as the Law of Economic Arrangements process continues without significant change, despite the ruling of this Court and the opinions of legal advisors to the government and the Knesset, the questions about judicial intervention in “what if” situations are likely to continue and increase, not in order to undermine the Knesset but in order to strengthen it and protect it from contempt for its vital work, which is important and necessary for the Israeli polity… ([43]: 4767).

Justice Rubinstein’s remark may imply that in the future the Court is likely to provide a different interpretation to the expression “a realistic opportunity to participate in the process,” which was coined in the Poultry Breeders ruling. The apparent tendency is to interpret this expression substantially rather than in a formal technical sense, thereby enabling the Supreme Court to exercise creativity in the sphere of public policy. The Court’s criticism as well as its willingness to indicate the need for change, though not enough to accept the petition, reflects an aggressive position from an institutional perspective. According to this position, the Court is willing to confront the Knesset and the Finance Ministry by using the rhetoric of protecting the Knesset and the public interest in it, and reinforcing its position to ultimately advance the institutional interests of the Court itself.

A third case that illustrates the institutional interests of the Court is the matter of the *Mayor of Ashdod* vs. *the Ministry of Finance* [35]. This was a petition by the mayor of Ashdod requesting the Court to instruct the Finance Ministry to refrain from advancing legislation in the context of the Law of Economic Arrangements that would cancel initiatives to build a hospital in Ashdod. The petition was made following a law that the Knesset had passed and a petition that had been granted to implement the law. The petitioner claimed that the Ministry’s legislative attempt would undermine the rights of Ashdod residents and thwart the efforts of over a decade to construct a hospital in the region. Through a hasty process and without any serious public debate, the Ministry was seeking to nullify a legislative action of the Knesset. The Court rejected the petition while noting the general ruling regarding judicial intervention in Knesset activity according to which the Court should refrain from intervening in an ongoing legislative process. Justice Beinisch, who drafted the ruling, added that in this case the petitioners had not provided a reason that would justify the rare intervention of the Court. In this case, the Court was unwilling to create friction with the Ministry or the Knesset, and it did not express an opinion regarding the processes by which the Ministry seeks to actualize its policy. Thus, the Court allowed the Ministry to play a dominant role in determining socio-economic policy even though this policy might have implications that deserve the Court’s considered attention.

More recently, the Supreme Court was asked to void two substantial amendments suggested by the Law of Economic Arrangements in the matter of the *Israeli Medical Association v. the Knesset* et al. [74]. This case involved a petition against a new section of the law that was about to go into effect in July 2016. The section included two important changes to the practice of private medicine in Israel. The first involved the revocation of the possibility of being reimbursed by the national healthcare funds or commercial insurers for private visits to any physician and instead mandated the establishment of a fixed list of physicians and surgeons who would be approved for this purpose. The second issue involved a prohibition to pay the healthcare provider directly and instead allow patients to pay the medical institution only, thereby constraining the commercial freedom of physicians offering private medical treatment.

Clearly, neither of these changes affects the state budget nor do they have any financial bearing on the healthcare system as a whole. These changes restrict the extent to which private medicine is practiced in Israel, and the means of enforcing these restrictions focus mainly on the freedom of occupation and contractual liberty of the providers themselves. Hence, one of the major arguments put forward by the petitioners related to the inappropriateness of the Law of Economic Arrangements to regulate these changes, especially when such changes have significant implications for the constitutional rights of the providers.

However, the Court, under the leadership of Justice Elyakim Rubinstein, decided to postpone its decision for six months and ruled that there was not yet much evidence pointing to the success of the new arrangement. The court held that it remained to be seen whether there would be data to support the petitioners’ claims regarding the violation of their constitutional rights, although it hinted that there was a great deal of doubt that such evidence would be forthcoming. The court did not indicate the kind of data it would be willing to consider in the future, nor did it explain why such data were necessary especially given the conceptual - as opposed to empirical - character of the claims suggested in the petition. Moreover, the court did not address the main argument put forward by the petitioners concerning the appropriateness of the Law to regulate these issues. However, it also did not accept the state’s position, leaving a void on this important issue. Both parties submitted their updated reports. In September 2017 the Court decided to allow the government to implement the proposed reform. While in principle, such a reform would strengthen the public healthcare system, increase access to services and reduce costs - outcomes that do not represent a neo-liberal approach at first glance - the court restated its tendency to accept the respondent’s claims about the weakness of the petitioners’ constitutional arguments but did not base its decision on such concepts. Instead, its decision reflects its choice to allow for the gathering of additional and more factual data that could have put aside the petitioners’ claims without having to protect a different ideology.

## Results

These four cases illustrate the phenomenon described in the previous sections in which a review of legislation based on the Law of Economic Arrangements on matters of healthcare allows the Court to strengthen its institutional political standing in its inter-relations with the Finance Ministry. The strategic position adopted by the Court is complex. In all of these cases, the Court did not overturn the Ministry’s policy by invalidating the Law. It allowed the Finance Ministry to be the first and foremost player in determining healthcare policy (including changing previous policy) and dictating the social and economic spectrum of values related to such policy. Nevertheless, the Court did not hesitate to criticize the steps taken by the Ministry on more than one occasion and recently commented on the extent to which the Law accords with specific constitutional material as well.

The Court’s unwillingness to interfere with or to issue an opinion on the subject matter of the various petitions presented to it, which, for the most part, present substantive claims against the Law, leave substantial arguments undecided. Under an institutional explanation offered here, it enables and reinforces a neo-liberal philosophy that sanctifies privatization in the healthcare system and the decline of the welfare state [75]. Such a philosophy may have far-reaching implications in terms of promoting the wellbeing of the general public, contributing to superficiality and silencing public debate on fundamental social matters, and undermining the public legitimacy of policymaking on matters that shape Israeli society.

## Discussion

Since the passage of the Law of Economic Arrangements, the percentage of private funding for national healthcare expenditures in Israel has risen from 26.2 to 36% in 2016 [76], and the government’s portion dropped from 74% in 1996 to 62% in 2017 [76]. The increase in the contribution of households to national healthcare expenditures was primarily the result of price increases in medications and services. However, some of these increases were also the result of the public’s purchase of additional healthcare insurance, which in 2005 represented over a quarter of the total expenditures of household on healthcare services and products. In a move that accords with the notion of alternative politics, this trend also encourages many Israeli citizens to seek and acquire healthcare services through alternative channels. The cumulative effect is a sense that the Israeli public prefers the supply of services provided by the private sector [77]. In addition, in many cases the government even encourages or subsidizes such channels as a response to apparent public demand, thereby increasing the perception that the private market is preferable to the public market when it comes to the provision of services [7].

A neo-liberal world outlook enforced by the Supreme Court for institutional reasons has also contributed to the erosion and lack of motivation to defend and protect the legal right to healthcare services. This right is not explicitly mentioned in the constitutional legal documents reflected in the Basic Laws. Therefore, the Supreme Court has ruled that although the right to basic healthcare services can be anchored in the right to bodily integrity constitutionally protected under Basic Law: Human Dignity and Liberty, it does not translate into an entitlement to membership in a national health insurance plan. The Court added that the National Health Insurance Law is only a mechanism intended to organize the provision of services to residents. Therefore, denial of membership in this mechanism should not be viewed as impinging on the rights of individuals to dignity and bodily integrity protected under the Basic Law [78]. In another case, the Court (per Justice Beinisch) noted that the minimal scope of the right to healthcare services is hard to define as it represents an all-encompassing collection of rights linked to human health, some of which enjoy constitutional status. Thus, in the Court’s opinion, the constitutional status of the right to healthcare services should not be examined as a single entity. Rather, the rationales behind the right and the interests protected by it should be examined in accordance with their relative social importance and based on their proximity to the constitutional rights enshrined in the Basic Law.

The Court stated that even if a constitutional right to public health services is found, the question arises as to how to interpret and apply the restricted ruling when there is evidence that this right has been violated. Justice Beinisch, however, refrained from ruling on the complex questions dealing with the constitutional status of the right to medical care generally and the right to healthcare services at public expense specifically [79].^15^ The Court’s unwillingness to discuss, declare or acknowledge the constitutional right to healthcare services in Israel was further upheld in later decisions [80–84]. In all these cases, the Court expressed a serious doubt as to whether there exits such a constitutional right. The Court rejected the claim that even if the right to heath care services can be derived from the constitutional right to human dignity or personal autonomy, it nonetheless does not entail a constitutional claim concerning the right to access a specific drug or medical procedure [84], the right of children to Israeli fathers whose parenthood has not been determined to be included in the universal coverage of health services [82] or to choose one’s healthcare provider [85, 86].

It follows that the Supreme Court has refrained from addressing the question of the constitutionality of the right to healthcare services. At most, and as with other social rights, it is willing to recognize the protection of the minimal essential services for elementary subsistence [87–89], as distinct from the revered status of the right to healthcare services in international law, which obligates Israel as well.

Indeed, from historical, analytical and legal perspectives, the right to healthcare services belongs to an advanced generation of socio-economic rights whose protection through judicial review is perceived as less justified than “traditional” “negative” rights. This is especially reflected in the constitutional law in Israel [90, 91]. It is argued that judges lack the democratic legitimacy to enforce social rights and the institutional capacity to do so (Landau, 2012; [92]). It is also argued that because of their large-scale consequences for the government’s budget, the judicial review of social rights results in remedies that impose more requirements on the state and on the allocation of social resources than the judicial review of civil and political rights such as the right to freedom of expression or human dignity. Therefore, according to this argument, social rights should not be enforced by courts and thus cannot be regarded as constitutional [93]. To this one should add that despite its authority to invalidate unconstitutional laws, the Supreme Court exercised such authority in a relatively small number of cases (less than 20 cases over the past 26 years) given the political debate on the legitimacy and existence of such authority. From this perspective, denying petitions regarding healthcare legislation should not be seen as a rare phenomenon in the field of judicial review of legislation. Moreover, the fact that the right to healthcare services is not specifically mentioned in the Basic Law: Human Dignity and Liberty and that proposals to legislate social rights, including the right to healthcare services as constitutional rights have been rejected in the past twenty years, *eg* proposal of Basic Law: Social Rights, by MP Ophir Pines-Paz, p/17/2864, dated 23.7.07, makes it more complicated for the Supreme Court to invalidate, by interpretation, Knesset legislation affecting this right.

Our analysis is not in contradiction to some other legal petitions in which the Supreme Court ruled against the Ministry of Finance and the State in cases where the respondents were asked to reconsider updating the health budget through the healthcare index, most notably Ruling 2344/98 [94] and Ruling 8730/03 [95]. Not only did these cases involve judicial review over administrative decisions, but they also did not include any substantial discussion of the right to healthcare and its status nor did they involve Economic Arrangement Laws and a direct institutional confrontation with the Ministry of Finance. These cases merely focused on the question of whether the respondents acted with reasonableness when they ignored the recommendations of the healthcare council to consider this update given the increasing health needs of the population and the costs of sickness funds which lead to erosion in the budget of the medical basket throughout the years. Moreover, in Rulings 8730, 10778/03 [95] there was even no dispute between the petitioners and the Ministry of Finance regarding the need to update the healthcare index. These cases called upon the respondents to act timely, consider and include all the relevant considerations in determining whether and if so, to what extent such a budgetary update is at place.

In sum, in Israeli law there is limited representation of the constitutional right to healthcare services and the protection afforded to it, both at the constitutional level and at the legislative level. The institutional political interests of the Supreme Court in its interaction with the Finance Ministry, particularly surrounding questions involving the Law of Economic Arrangements, fuel and are fueled by a neo-liberal perspective that preserves, and at times even promote, the relative institutional standing of the Supreme Court. However, this outcome may also lead to the erosion and weakening defense of the right to healthcare services. Furthermore, it does not prevent the phenomena of the privatization of the healthcare system and the decline of the welfare state.

## Conclusions

The Law of Economic Arrangements is a political mechanism through which the Finance Ministry seeks to create, alter, and eliminate public policies, including policies set by the legislative authority itself. By approving this legislation, even in the face of the serious flaws in the legislative process and the effects of such legislation on the right to healthcare services as well as on other socio-economic rights, the Israeli Parliament is acting against the democratic perspective intended to guide it. Judicial review of such legislation does not constitute a review of a traditional autonomous function of the Parliament but of activities that diverge from the democratic political system.

Nevertheless, an institutional analysis of the Supreme Court rulings concerning this Law reveals that the Court has an institutional political interest in increasing the public’s trust in itself, at times by taking a position that clashes with the Finance Ministry bureaucrats. Aside from exercising judicial restraint and allowing more leeway to the executive and legislative branches more generally, the specific explanation offered for this clash allows the Court to be a dominant and central player in the formation of public policy. These minor frictions with the Ministry of Finance can also be explained by recent institutional changes following the social protests in Israel in 2011. These changes reflect a possible shift towards participatory democracy and deliberative decision-making [96], imposing on the Parliament the obligation to conduct the legislative process so that it is more transparent and involves the participation of various segments of society. However, in an environment in which social rights have a relatively weak status, the Court is reluctant to intervene through a judicial review of the right to healthcare services that is not specifically included in the constitution. We maintain that this enabling strategy of the Court allows for the promotion of values identified with neo-liberal philosophy. Furthermore, it does not prevent socio-economic phenomena that amount to a decline in the welfare state, the privatization of the healthcare system, and the undermining of the legal right to healthcare services. Most of all, it provides a blatant path to depart from the principles of justice, equality, and solidarity that underpin Israel’s healthcare system and that are the basis of the National Health Insurance Law.

While the problem of non-governability characterizing Israeli politics highlights the hidden interaction between the judiciary and the executive through the unique model of the Law of Arrangements most effectively, our analysis can be generalized to other political systems where the courts seek to increase their power and promote a right-wing ideology, yet appear neutral and objective. In such situations, they may choose various courses of action including those that support an opposite philosophy. Theoretically, our analysis provides strong support for our claim that although bureaucrats and courts may have had a general policy of reducing government intervention in the economy, under conditions of non-governability most of their activities and initiatives with regard to the healthcare system will involve hidden interaction between the judiciary and the executive rather than being the result of long-term strategic plans to retrench the welfare state.

Moreover, unlike many of the discussions in the literature regarding judicial review as a legitimate forum for resolving the competing interests of the Court and the Parliament with the former having a negligible influence on politics and policy, we demonstrate how under one explanation which is the subject of this article, the Supreme Court seeks to increase its institutional power at the expense of the Government in an era of non-governability. As our article shows, the various outcomes that support this conclusion reflect a real departure from what is currently discussed within Israeli politics and invites further investigation as to the complex relationship between the courts and the government, and the effect of such actions on the formulation and implementation of public policy.

## Additional files

Additional file 1: Judicial review of the Knesset. (DOCX 25 kb)

Additional file 2: Judicial review on budgetary and economical maaters. (DOCX 24 kb)

## Footnotes

1 Because non-approval of the Budget Law three months after the start of the fiscal year amounts to a decision to dissolve the Knesset, passing the law is guaranteed. When the bill is not linked to the Budget Law, the government announces that it regards voting for the law as a vote of confidence, thereby guaranteeing its approval. See, for example, High Court of Justice Ruling 4885/03, Poultry Breeders Union in Israel vs. the Government of Israel, Ruling 49 14(2) (Hebrew).

2 Throughout the years there has been an increase in the number of issues regulated by the Law of Economic Arrangements and a dramatic rise in the number of provisions in the law. For a comparative review, see [97].

3 In the past, all of the issues of the Arrangements Law were transferred to the Finance Committee. In recent years, some of the issues have been transferred to the Finance Committee (at the request of the Government), while some of them were transferred to the other Knesset committees for discussion and decision, according to their areas of activity. Nevertheless, it is the Finance Committee that presents the bill as a single unit for a second reading and third reading in the Knesset. To pass a bill in the Knesset, it must be voted upon three times.

4 Although recently, and following criticism of the Law of Economic Arrangements procedure, the bill has been divided up for debate among the various Knesset committees, most of the bill is still debated only in the Finance Committee [98] and after it is approved on the first reading and discussed in one of the Knesset special committees, it is forwarded to the plenum as one whole piece for approval on the second and third readings [99].

5 Note that while politicians play important role in setting the budget, economic policy and health policy and they may also narrow the range of options available to bureaucrats, in Israeli politics the Ministry of Finance generally, and the budget division specifically, has a great deal of power and political standing that surpasses that of other countries that are members of the OECD [100].

6 For exceptions with regard to recognizing the Court’s authority to intervene, see Additional file 1.

7 There is an argument that the Law of Economic Arrangements is a law (or norm) situated below a regular law in the pyramid of norms, and therefore it cannot amend laws of a constitutional nature. There is debate about this position (Golan, 2007–8: 267). In this article we regard the Law of Economic Arrangements as having the same status as a regular Knesset law.

8 For the legal rules on this notion, see Additional file 2.

9 The Court observed that the advantages of the Law in terms of efficiency do not outweigh the principle of the separation of powers and the principles of representative democratic rule. Therefore, any use of this Law should be informed and limited ([29]: 35–6). In the Court’s opinion, the fact that the Knesset has the authority to undertake a legislative process along the lines of the Law does not mean that there would never be a place for judicial intervention in the legislative process ([29]: 41–2).

10 This transformation was primarily facilitated by the school of thought developed by John Rawls ([101]: 135–6).

11 This is also the reason, for example, that advocates of the strict libertarian perspective, such as Robert Nozick, object to the notion of taxation as a means of promoting social welfare.

12 Compare the words of Aharon Barak, who until 2006 was the president of the Supreme Court, according to which “human dignity is not a socialist program” ([102]: 419).

13 Compare the High Court of Justice Ruling on the Council for Citrus Fruit Marketing [33].

14 In particular the Court referred to the principle that intervention in legislative processes should occur when there is substantive harm to the principle of Knesset members’ participation.

15 In a later Supreme Court decision in the matter of Davidov, the argument was again put forward that not including life-saving medication violates the constitutional right to healthcare services. The Supreme Court referred to Luzon and stated that the question of whether a constitutional right to healthcare services exists had not yet been settled [80].
