# Supplementary material for: A neo-institutional analysis of the hidden interaction between the Israeli Supreme Court and the Ministry of Finance: the right to healthcare services
Source: Isr J Health Policy Res. 2018 Nov 27;7:71. doi: 10.1186/s13584-018-0261-9 (PMC6258264; doi:10.1186/s13584-018-0261-9)
Supplement: Supplementary file 1 — Judicial review of the Knesset. (DOCX 25 kb) [file 13584_2018_261_MOESM1_ESM.docx]

Appendix A

There are rationales for making an exception with regard to recognizing the Court’s authority to intervene. These rationales might be tied to circumstances where Knesset members do not have “a realistic opportunity to participate in the process.” Such a lack of opportunity undermines the principle of participation, making a judicial review necessary to defend and ensure that the limitations of the Knesset are not exceeded (Ruling 266/68). Additional circumstances that justify intervention in Parliamentary action exist when “final” outcomes are created and criticism *post-factum* cannot be handled effectively (Ruling 761/86: 876). Similarly, such intervention is justified when the legislative process involves a defective debate that affects the essence of the process (Ruling 2836/03). In other words, intervention can occur when there is a violation that undermines the fundamental values of democracy that are the basis of the legislative process (Ruling 5131/03). These are the values of the State of Israel as a Jewish and democratic state. They include the principle of representation, the principle of implementing the will of the majority (formal democracy), and the principle of equality, freedom of expression, and public faith in the Knesset (essential democracy) (Ruling 5131/03: 588). However, according to the Supreme Court, only in the case of a breach of these values leading to severe and pronounced impact will the Court be ready to nullify the law (Ruling 7610/03: 4). This reluctance allows the Knesset to maximize all of its political forms of expression by creative a normative reality, where judicial intervention would be superfluous (Ruling 761/86). On this matter, the Court may use the principle of proportionality, seeking to determine compensation for the petitioner (Ruling 5131/03: 589). The Court also determined that in addition to the profound and substantial impairment of the Parliamentary procedure and the extent of damage to the fundamental principles of democracy, the question of whether the law might have been adopted without the flaw should be assessed. Furthermore, the reasonable expectations that it created as well as the consequences of nullifying the law should also be considered (Ruling 4885/03: 42-43). Finally, when a petition to the Court asserts that a legislative amendment conflicts with the principles of the Basic Laws (Israel's constitutional framework), the petitioner might argue that a hasty legislative process, like that characterizing the Law of Economic Arrangements, does not allow a thorough public debate regarding a fundamental question. Therefore, it is a disproportionate means for amending the law, thereby violating constitutional rights beyond what can be permitted (Basic Law: Human Dignity and Liberty: §8).

Bibliography

High Court of Justice Ruling, 266/68 The City of Petah Tikva vs. the Minister of Agriculture, PADI 22 (2) 824.

High Court of Justice Ruling, 761/86 Mohamed Mi’ari vs. the Speaker of the Knesset, PADI 42 (4) 868, 873.

High Court of Justice Ruling 2836/03, Mayor of Ashdod Mr. Zvi Zilker vs. the Ministry of F High Court of Justice Ruling, 5131/03 Yahadut Hatorah vs. Speaker of the Knesset, PADI 59 (1) 577, 586.inance (Nevo).

High Court of Justice Ruling, 7610/03 The Regional Council of Yanukh-Jat and Members of the Council et al. vs. the Minister of Interior (Nevo).

High Court of Justice Ruling, 4885/03, Poultry Breeders Union in Israel s. the Government of Israel, PADI 49 14(2).
