# Supplementary material for: A neo-institutional analysis of the hidden interaction between the Israeli Supreme Court and the Ministry of Finance: the right to healthcare services
Source: Isr J Health Policy Res. 2018 Nov 27;7:71. doi: 10.1186/s13584-018-0261-9 (PMC6258264; doi:10.1186/s13584-018-0261-9)
Supplement: Supplementary file 2 — Judicial review on budgetary and economical maaters. (DOCX 24 kb) [file 13584_2018_261_MOESM2_ESM.docx]

Appendix B

In a number of cases, the Court ruled that making budgetary decisions about social goods is a political activity and should be made by representatives of the public (Ruling 4947/03). Therefore, judicial intervention is not legitimate (Reichman, 2004: 314-5). Moreover, decisions about such issues require expertise and familiarity with the entire budgetary picture. Such characteristics are within the purview of the budgetary personnel, not the Supreme Court. Arguments have also been made that the Court lacks the institutional ability to make a decision on matters with budgetary implications because the adversarial process itself does not allow the Court to examine issues in the long term and in their full context (Reichman, 2004: 314). Furthermore, its perspective is retroactive and specific for the most part (Davidov, 2008: 351). In the context of healthcare, due to the controversy about the nature of the State's obligation to promote healthcare as a social good under international human right law and the lack of clarity regarding the normative meaning of the right to healthcare, one can argue that this issue is not justiciable. Because of the difference between this issue and other matters that the Court hears, case law on the right to healthcare largely confines itself to issues concerning the minimum content of this right and to the State's basic obligations in this regard. Therefore, the State has a great deal of discretion with regard to the remaining components of the right to healthcare (Hendrix, 1998).

On the other hand, some argue that when budgetary decisions undermine the human rights that are anchored in law, such as the right to healthcare determined by medical needs, the Court should exercise judicial oversight and intervene (Shalev, 2001). Further justification for this intervention is rooted in the fact that healthcare does not function like a regular market with clear rules (Phelps, 2003). A related approach seeks to enable the constitutional review of legislation that has substantial budgetary significance and strikes at the “core” (as opposed to the “periphery”) of a constitutional right. Davidov (2008) describes this situation as that which is at “the core of what, given its importance, the judicial system is intended to protect at all cost” (p. 382). A different approach would accept the use of judicial review in situations where it is evident that the interests of a weak, minority, or marginalized group in society have not been adequately represented (Ely, 1980). It can certainly be argued that the hasty legislative process to pass the Law, the fact that most Knesset members vote in favor of the Law having no information about or independent opinion on its content (Golan, 2007-8: 263), as well as the lack of opportunity for a broad participatory debate that reflects the range of voters' representatives undermine this important principle. At the very least, an internal constraint can be included to defend the public’s social rights. For example, the Court could stipulate that the actualization of the relevant right would depend on the economic ability of the state as determined by the government. Similarly, it could require that the enforcement of the rights could be conditional on their being reasonable and not creating social gaps inconsistent with the state’s objectives as a Jewish and democratic state.

Bibliography

High Court of Justice Ruling, 4947/03 The City of Beer-Sheva vs. the Government of Israel Supreme Court, Cases 2006 (2) 1545.

Davidov, G. 2008. Constitutional Review in Budgetary Matters. Mishpatim 49: 345 (Hebrew).

Ely, J.H. 1980. *Democracy and Distrust: A Theory of Judicial Review*. Cambridge: Harvard University Press.

Golan, T. 2007-8 The Democratic Deficit of the Law of Economic Arrangements and the Erosion of the Israeli Welfare State. *Law and Government* 11: 247 (Hebrew).

Hendrix A. 1998. The Right to Health in National and International Jurisprudence. *European Journal of Health Law* 5: 389-403

Phelps C. 2003. *Health Economics*, 3^rd^ ed. Boston: Addison Wesley

Reichman, A. 2004. The Democratic Deficit and Constitutionality of the Economic Plan. Labor, Society and Law 10: 314-315. (Hebrew).

Shalev C. 2001. National Health Insurance: Update of the Basic Medical Basket Medicine & Health 25: 190 (Hebrew)
